# On the Influence upon Health, on the Introduction of Tea and Coffee, &c.

**Published:** 1849-08

**Authors:** 


*On the Influence upon Health, of the Introduction of Tea and Coffee in large proportion into the Dietary of Children and the Laboring Classes.* By SAMUEL JACKSON, M. D., Professor of the Institutes of Medicine, in the University of Pennsylvania.

Tea and coffee enter more largely into the diet of the people of this country than into that of any other. The ordinary breakfast and supper of thousands of persons in every part of the United States, are tea, coffee, and bread; while tea, bread, and potatoes, with occasionally a modicum of meat, constitute their dinner. Even children, as soon as they are able to sit at meals, are habitually placed at the family table, and allowed to partake of the same food as adults.

In the poorer classes, the evil of a common diet for all ages, cannot probably be avoided. It is one of the causes productive of the greater mortality of the children of the poor. But *this* injurious practice, which with the poor is to be regarded as an inevitable misfortune, is followed by those who are placed in circumstances above the necessity of it. In them it is most condemnable, and can be excused only on the plea of ignorance.

The classes in which the kind of alimentation alluded to prevails, are female teachers, seamstresses, factory women, weavers, tradesmen, small retailers, clerks with families, and others living on restricted means, and very generally farmers in the country.

The inducement for its adoption is its economy, as to money, time, and fuel — a meal of coffee, or tea and bread, or the addition of potatoes, for a small family, will not cost beyond a few cents, while it requires but little fuel, and a very short time for its preparation. Tea and coffee are, besides, very palatable, produce temporary exhiliaration and force, and abate hunger. Coffee, as will be shown, is not devoid of some nutritive properties. Ostensibly answering, in this manner, the purposes of food, tea and coffee have, from the considerations of cheapness and convenience, become the substitutes of more substantial diet.

In this country and England, chiefly, tea and coffee are introduced into the daily meals as aliment. In China, tea is used as a refreshing and cordial beverage, presented to visitors, or drunk between meals; in the East, coffee is regarded in the same light, and employed in the same manner; on the continent of Europe, coffee is extensively used, but more as a cordial drink, or to flavor cream and milk, than as aliment.

In prosecuting this inquiry with a view to the effects on the economy, of tea and coffee, some preliminary matters require a previous examination.

Every one knows that food is indispensible to life. But what is this connection between them? How is it that food is an indispensible condition of life? The solution of these questions is necessary to the understanding of the nature and objects of food, to determine the value of any alimentary articles, and to settle the pretensions of any substance for a place in the category of food.

Before examing the relation existing between food and life-action, it is important to obtain an accurate idea of what is life, or organic action. This term we limit to a single series or class of phenomena. These phenomena are the evolution or production of specific organizable matter and definite organic forms, from a primary formless organic substance. Albumen is that substance in man and the higher animals. All other phenomena are excluded. They are subordinate to, depend on, but are indispensible to maintain life-action.

Organized tissues and organs worked out by life-action, are the instruments of life. They differ widely from each other. Each has its special office. The phenomena of each are special in character and purposes. They are the same as similar phenomena in the exterior and inorganic world. They can be properly understood and studied only in their connection with those phenomena. Some are chemical, as the transformation of albumen, the processes of digestion, secretion, and the oxidation of carbon and hydrogen in the blood producing animal heat. Others are physical, as the capillarity of tissues, imbibition, endosmose, atmospheric pressure, and Graham's law of the diffusion of gases, in respiration; others are dynamic, as the excitor, motor, and other forces of the nervous system; others, again, are purely mechanical, as the actions of the muscular system.

Not one of those is properly an organic or life-phenomenon. They are indispensible to maintain the condition of the existence of life, or organic action. They are chemical, physical, dynamic, and mechanical actions, executed by organized and living apparatus and instruments, for the objects of life.

The organizable matter and organic forms are the products, and, consequently, the expression of existing forces or causes of action. Forces, matter, and form are indissolubly connected with, and give rise to phenomena or function; and, inversely, function and phenomena are the correlatives of force, matter, and form. Organized matter, from its nature, cannot be persistent. Under normal states, force, matter, form, and function or phenomena, are permanent; but the structural material itself is not permanent—it wastes, decays, disintegrates, and is reproduced in every act of life. Life-action is thus resolvable into two inseparable actions, or links of one action, a birth and a death, the formation and destruction of the organic material of our structnre.

The supply of the primary organic substance for this incessant renewal and building up of the organized structure and maintenance of organic forms, is derived from the blood. This fluid, in its natural state, is a concentrated solution of all the solids and products of the animal economy. The amount of azotized or albuminous compound matter destroyed in 24 hours by life, or organic action, may be taken, on an average, at two to three ounces.* The blood would rapidly become impoverished and unfitted for life objects, unless its losses of albumen and its organic derivatives were constantly restored, The renewal of the organizable or plastic material of the blood, and its maintenance in its normal composition, for structural formation, is one of the offices of our food. Repeated analyses have demonstrated that, of the aliment that is adapted to healthy nutrition, one-eighth part only consists of albumen, or its protein compounds, or their derivative compounds; and whatever is devoid of those substances—that is, the chemical combination of carbon, hydrogen, nitrogen, and oxygen, in the proportion to constitute protein ($C_{40}H_{30}N_5O_{12}$†) or albuminous compounds—cannot perform the office of food, or be fitted for nutrition.

Another condition, not less indispensible to life-action, than organizable or plastic matter in the blood, is a definite temperature. For man and the warm blooded animals, the heat essential to healthy, vigorous life-action is 98° to 100° F. So important is heat to life, that nature has made a provision for its constant disengagement in the economy. This is accomplished by the incessant oxidation of carbon and hydrogen in the blood. The temperature of this fluid is thus kept at an equable point in every part of of the economy. Every organized molecule requires, for the exciting and sustaining of its life-action, the presence of plastic or organizable material, and a definite temperature.

The blood furnishes both these indispensable conditions of life-action to each living molecule.

The carbon and hydrogen oxidized in the blood, and in this manner generating animal heat, are obtained from the food. Nature has made most ample provision for the supplies of these chemical elements, by constituting them a large portion of the food of animals. Not less than from

*Dumas, Chimie Phisiologique et Medical, p. 463. †Mulder.

six-sevenths to seven-eighths of the alimentary substances of animals consist of non-azotized bodies. Fatty, starchy, and saccharine matters, are of this character; they are not adapted to, or intended for nutrition, but solely for the purpose of calorification, by their combustion or combination with oxygen introduced into the blood by the processes of respiration. This proposition is demonstrated in the composition of the alimentary portion of milk. The casein or plastic matter for nutrition, averages 13 per cent., the calorifacient, or the cream and sugar of milk, 87 per cent.

Temperature is required not only for life-action, but also for the dynamic forces, and mechanic power and actions seated in and performed by the muscular apparatus.

The identity of heat and mechanic force has been established by M. Joule.* It is expressed in the following formula: the heat required to raise one gramme (15 grains) of water one degree (cent.), is capable of raising 432 grammes (3700 grains) one metre, or 3½ feet.

According to the estimate of Dumas, the quantity of carbon consumed by a man in good health (valuing the hydrogen by an equivalent proportion of carbon), averages from seventeen to twenty-eight ounces per diem. The large amount of heat thus disengaged, is the sum of the dynamic or excito-motor force of the nervous system.

By the establishment of the above facts, we obtain precise ideas of the nature of food, its objects in the economy, and the modes of its operation. We are enabled to say with certainty what substances are, or are not food; and to fix the relative value of each article of diet.

From these investigations, it is ascertained that alimentary substances form two distinct classes, differing from each other by the most striking diversities of nature, composition, and operation.

The first class are the protein or albuminous compounds. They have nearly the same chemical compositions as the tissues, are isomeric with many of the immediate organizable materials of animal structure, and are exclusively destined to nutrition proper, or the reconstruction and repair of the solids.

No substance in which this especial chemical composition, protein and its compounds do not exist, can belong to this class, or can be employed in the economy for its nutrition. Some of the most eminent organic chemists and physiologists appear to suppose, that any organic nitrogenized body may answer for nutrition. This is not so. Morphia. quinia, strychnia, urea, taurine, as well as theine, and caffeine, are organic nitrogenized bodies, yet cannot be ranked as food. It is the possession of the specific combination of which protein is the base, that can alone entitle any substance to rank in this class.

The second or calorifacient class of aliment, comprehends those special chemical compounds, hydro-carbons mostly, that are capable of prompt decomposition into carbon and hydrogen in the blood. No other organic substances, though rich in carbon and hydrogen, are capable of entering into this division of aliments.

The normal substances of this kind are glucose and lactic acid, into which saccharine and amylaceous substances are converted by the process of salivary digestion; and fatty matters, modified and reduced to the finest

*Comptes Rendus, tome xxv. p. 209.

and minutest particles possible, in the emulsion formed with them by the pancrealic and biliary secretions.

This last class is the more immediately connected with the maintenance of life. It is established by the experiment of Chossa,* that death from starvation does not occur from inanition, or the waste of the organs, but from the cooling of the blood, from the absence of the carbon and hydrogen requisite to carry on the process of combustion and the generation of caloric.

With the preceding facts ascertained, we can now proceed to investigate the claims of tea and coffee, to be regarded as properly belonging to either of the above classes of food.

Theine and caffeine, according to Leibig, are the essential elements of tea and coffee. The two are identical as to chemical equivalents. The formula for each is $C_8,H_5,N_2,O_2$. M. Payen, in a later and more elaborate examination, gives a somewhat different formula, but not such as to vary their properties to any extent. Liebig considers them as closely approximating to alloxan, $C_8,H_4,N_2,O_2$, a principle obtained from urea, by the action of concentrated nitric acid; and to taurine $(C_4,H_8,NO_{10})$, a principl which may be obtained from ox bile, but not from human bile.

In this view, tea and coffee must be excluded wholly from the classes of aliments, to which theine and caffeine can have no pretensions.

But M. Payen, in 1846, in a communication to the Academie des Sciences,† presented a highly labored and accurate examination of the proximate constituents of coffee, which unquestionably brings it, at least, into the category of aliments, as it contains the constituents of both classes.

The following is his analysis of coffee:‡

| | |
|---|---|
| Cellulose, | 34 |
| Fat substances, | 10 to 13 |
| Glukose, dextrine, and an indeterminate vegetable acid, | 15.5 |
| Legumin, casein, (gluten), | 10 |
| Chloroginate of caffeine and potassa, | 3.5 to 5 |
| Azotized organic matter, | 3 |
| Free caffeine, | 0.8 |
| Insoluble concrete vegetable oil, | 0.001 |
| Fluid aromatic essence of sweet odor, and a less soluble acrid aroma, | 0.002 |
| Mineral substances, potassa, lime, magnesia, phosphoric, sulphuric, silicic acids, and a trace of chlorine, | 6.697 |
| Water, | 12 |
| | 100 |

From this composition of coffee, it is evident the grain is endued with nutrient or plastic and calorifacient elements, and, consequently, is an aliment; yet the proportion of those elements is not sufficient to place it in a high rank in either class, or to justify the substitution of its infusion as a

*Recherches Experimentales sur l'Inanition, Paris, 1843.

†Comptes Rendus, tomes xxiii., 1846.

‡Comptes Rendus. Tome xx[illegible] [illegible]49.

chief material of food, by those who are engaged in active and laborious pursuits.

But when the quality of the weak infusion almost generally used as food, and the consequent very small proportions of the alimentary elements held in solution in it are taken into consideration, the disparity between the waste of the blood and the elements for its reparation contained in coffee, become strikingly displayed. The ordinary coffee of the laboring classes, is little more than warm water colored and aromatized by coffee. It contains but a very small portion, if any, of the nutritive and calorifacient elements. It is impossible, with such diet, to maintain in the blood the two indispensible conditions of life-action and nerve-force, organizable material and heat.

Coffee, to be prepared as food, should be first but slightly roasted, merely browned and rendered crisp, so as to be easily reduced to a coarse powder. A concentrated infusion is then to be made by the process of displacement. There should be added to it an equal or double its quantity of cream or good milk, and be sweetened with sugar. An alimentary drink is thus prepared, possessing all the requisites of good food, with the addition of a specific excitant action on the nervous system and brain, that entitles coffee to the appellation bestowed on it by Rousseau, "boisson intellectuelle."

The ordinary miserable preparation of coffee is so extensively used as food, deficient in proper alimentary principles, by taking away appetite, by distending the stomach with a warm liquid, and thus impairing its digestive power, and by its agreeable aroma corrupting the taste, rendering more nutritious food unpalatable, tends to the ultimate impoverishment of the blood. This fluid loses its proper character, that of a concentrated solution of all the organic elements and products of the economy.

As a consequence of this condition of the blood, the waste of the tissues exceeds the repair, death-action is stronger than birth-action; disintegration of structure predominates over its reformation. In time this loss of balance tells: the organs are degraded from their primitive type; their functions are impaired, and the organism descends in the scale of development. There is an approach to inferior organisms, and to cold blooded animals; or, rather, the system is kept permanently in what constitutes the cold stage, or tendency to collapse in febrile diseases.

In this state, individuals suffer from a variety of vague anomalous symptoms, characterizing no definite disease. They are always ailing, complaining, suffering, but not absolutely sick. They are miserable themselves, a plague to doctors, the prey and victims of quacks.

In this condition of the economy, the temperature is low. Dynamic force, which is identical with heat, is equally depressed; and, consequently, the mechanic or muscular power is at zero, and the offices of the economy depending on it are imperfectly performed. The circulation is feeble, digestive movements slow and defective; languor and exhaustion prevail. Exercise augments the evils by expending the forces more rapidly than produced, and the nervous functions are in a state of perturbation and depression. These disordered states are the results of a slow inanition or starvation, not suspected, because food is taken to the full repletion of the stomach; yet still it is starvation, for the blood does not possess the elements for heat and nutrition adequate to the full energy and the consumption of life-action. These cases are not remediable by medicine; they can be relieved only by a restoration of the digestive functions, and a return to a wholesome and appropriate diet.

Cases of this character have augmented in our towns and cities, and it is believed in the country, particularly amongst women, and in the industrious and laboring classes, in the last ten or fifteen years most rapidly. The neuroses, as gastralgia, different visceralgias, and other forms of neuralgia, are now quite as common amongst those classes, if not more so, than they were formerly amongst the luxurious and idle, to which they were almost exclusively confined.

A suspicion has arisen that this circumstance is to be attributed to the perversion of the use, as food, of tea and coffee, from their proper employmont as nervous excitants and cordials, which are their appropriate properties. On inquiry it is almost universally found, at least, in the observations of many medical practitioners, that the greatest sufferers from these disordered states, are the inconsiderate consumers of tea and coffee, who substitute them largely for food.

It would extend this inquiry too far to enforce the above views by relations of specific cases. A large number could be cited as strongly illustrating their correctness.

The practice of giving tea and coffee to children at their meals cannot be too strongly reprehended and discountenanced. In the first periods of life, the most nutritive food, rich in plastic elements and capable of favoring the highest organization, is that which is required for growth and development. In the first fifteen years, nature is employed in constructing and perfecting the mechanism of life, fitting it for the conflicts, the exertions, the labors it must encounter and undergo in the struggles and difficulties of the great arena of the world, as well as with exterior malignant influences hostile to its existence, to which it is incessantly exposed. Without good materials, there cannot be produced a good fabric.

Whatever tends to excite, to render irritable, or to develop unduly the cerebral structure and functions in childred, is of necessity injurious. The bills of mortality show the fearful ravages in the early years of life from cerebral disease; and the foundation of most of the neurotic diseases and of ill health in adult life, dates from the abortive efforts of nature to build up substantial organs from the paucity and poverty of the building materials, or the abnormal direction imparted to nutritive action, by over excitement, in the commencement of development.

Tea and coffee being cerebral excitants cannot act otherwise than injuriously on children, in whom there exists no object for such artificial stimulation. Indirectly, they are mischievous by taking the place of food that contains all the elements and constituents of the fluids and solids of the organs and their products. They should be abolished from the dietary of children in all well-regulated families, and by parents careful of their children's welfare.

The analysis of tea is not complete, like that of coffee, by M. Payen. As far as known, it containt no alimentary elements, and cannot be classed with food. It is a purely cerebral excitant.

Though the grain of coffee has amongst its constituents alimentary elements, yet in the common slovenly process of torrefaction, the calorifacient principles are destroyed; and the plastic are also more or less decomposed.

But when more carefully performed, and these principles are not materially injured, still a small portion only can be dissolved in the infusion or decoction made in the ordinary mode.

The infusions of tea and coffee cannot, therefore, be used as food, and be made substitutes for nutritious aliment, without a serious detriment to the economy. They are cordial beverages, and as such are grateful and useful, especially to those engaged in mental pursuits, and who lead sedentary lives. They must, at the same time, be combined with substantial nutriment, or the blood becomes impoverished, and fails to contain the materials for organic structure, evolution of nerve-force.

In proportion to the degree of physical exertions, are the wear and tear of the solids, and the expenditure of the forces. The elements to maintain these in their normal conditions must exist in the blood, and the blood obtains them from the aliment in which they exist, through the digestive apparatus. Tea and coffee largely drunk at their meals by those engaged in active and laborious pursuits, by excluding a due quantity of substantial food, rich in the plastic and force-producing elements, are more injurious to these classes than to the sedentary.

The inevitable consequences of this practice must be to undermine the constitution, to impair the health, to break down the forces, to cause various nervous sufferings, and finally to produce disability for labor.—*Amer. Journal of the Medical Sciences.*

